# TLR Signalling Pathways Diverge in Their Ability to Induce PGE_2_

**DOI:** 10.1155/2016/5678046

**Published:** 2016-08-18

**Authors:** Valentina Salvi, Xenia Vaira, Veronica Gianello, William Vermi, Mattia Bugatti, Silvano Sozzani, Daniela Bosisio

**Affiliations:** ^1^Department of Molecular and Translational Medicine, University of Brescia, 25123 Brescia, Italy; ^2^Humanitas Clinical and Research Center, 20089 Rozzano, Italy

## Abstract

PGE_2_ is a lipid mediator abundantly produced in inflamed tissues that exerts relevant immunoregulatory functions. Dendritic cells (DCs) are key players in the onset and shaping of the inflammatory and immune responses and, as such, are well known PGE_2_ targets. By contrast, the precise role of human DCs in the production of PGE_2_ is poorly characterized. Here, we asked whether different ligands of Toll-like receptors (TLRs), a relevant family of pathogen-sensing receptors, could induce PGE_2_ in human DCs. The only active ligands were LPS (TLR4 ligand) and R848 (TLR7-8 ligand) although all TLRs, but TLR9, were expressed and functional. While investigating the molecular mechanisms hindering the release of PGE_2_, our experiments highlighted so far oversight differences in TLR signalling pathways in terms of MAPK and NF-*κ*B activation. In addition, we identified that the PGE_2_-limiting checkpoint downstream TLR3, TLR5, and TLR7 was a defect in COX2 induction, while TLR1/2 and TLR2/6 failed to mobilize arachidonic acid, the substrate for the COX2 enzyme. Finally, we demonstrated the* in vivo* expression of PGE_2_ by myeloid CD11c^+^ cells, documenting a role for DCs in the production of PGE_2_ in human inflamed tissues.

## 1. Introduction

PGE_2_ is the predominant eicosanoid produced in inflamed tissues and by growing tumors, with a major contribution by infiltrating immune cells [1, 2]. Because PGE_2_ promotes vasodilatation and accumulation of proinflammatory cells, it is generally recognized as a mediator of active inflammation. However, by suppressing the production of some proinflammatory cytokines, PGE_2_ also limits nonspecific inflammation and fosters the immune suppression associated with chronic inflammation and cancer [1, 2]. Despite the fact that PGE_2_ targeting is easily done by common and effective pharmaceutical agents (i.e., steroids and nonsteroid anti-inflammatory drugs), an accurate understanding of PGE_2_ regulation and mechanisms of action is crucial to fully deploy the therapeutic potential of these drugs.

The inflammatory synthesis of PGE_2_ is regulated by three classes of enzymes: cytosolic phospholipase A_2_ (cPLA_2_) family members that mobilize arachidonic acid (AA) from cellular membranes, cyclooxygenases (COX1 and COX2) that convert AA into PGH_2_, and specific synthases accounting for the final conversion of PGE_2_ [2]. While COX1 is housekeeping gene governing homeostatic PGE_2_ production, COX2 is potently induced by proinflammatory stimuli [3]. In inflammation, the rate of PGE_2_ production largely depends on the expression and activity of COX2, although it can be affected by other factors such as local availability of AA [2].

Dendritic cells (DCs) are professional antigen presenting cells responsible for the activation of the adaptive immune response [4] and also play a crucial role in the regulation of inflammation [5, 6]. For doing this, DCs are equipped with the vastest repertoire of pathogen-sensing receptor (pattern recognition receptors, PRR) such as NOD-like receptors, C-type lectin receptors, and Toll-like receptors (TLRs) [7–10].

Human TLRs are a family of type I transmembrane proteins [11]. Upon microbial recognition, TLRs recruit a specific set of adaptor molecules, such as MyD88 and TRIF, to initiate downstream signal transduction pathways. MyD88 is used by all TLRs except TLR3 and activates the transcription factor NF-*κ*B and mitogen-activated protein kinases (MAPK) to induce inflammatory cytokines. By contrast, TRIF is used by TLR3 (and TLR4) and induces the secretion of type I interferons and also some NF-*κ*B-depending genes [11, 12]. The TLR signalling cascades have been described using murine cells from knockout animals or immortalized cell lines of tumor origin. As a result, little is known about the pathways and cellular responses activated by TLRs in human primary cells.

Given the importance of PGE_2_ in the orchestration of the immune and inflammatory responses, we set out to dissect the molecular mechanisms underlying its release by TLR-specific ligands in human DCs.

## 2. Materials and Methods

### 2.1. Cell Preparation and Culture

Buffy coats were obtained through the courtesy of the Centro Trasfusionale, Spedali Civili, Brescia. Monocytes were purified from peripheral blood mononuclear cells (PBMC) by immunomagnetic separation using anti-CD14-conjugated magnetic microbeads (Miltenyi Biotec, Bergisch Gladbach, Germany). DCs were differentiated from monocytes cultured for 6 days in tissue culture plates in RPMI 1640 (Gibco, Life Technologies, Carlsbad, CA, USA) supplemented with 10% heat-inactivated fetal calf serum (FCS, Lonza Group, Switzerland), 2 mM L-glutamine, antibiotics (Gibco) (complete RPMI medium), 50 ng/mL GM-CSF, and 20 ng/mL IL-4 (ProSpec Technogene, Israel) as previously described [13]. Myeloid DCs (mDCs) were isolated using the CD1c (BDCA-1)^+^ Dendritic Cell Isolation Kit (Miltenyi Biotec).

### 2.2. Reagents

DCs or mDCs (2 × 10^6^ cells/mL) were stimulated with the following TLR ligands: 100 ng/mL PAM_3_CSK_4_, ligand for TLR1/2; 100 ng/mL FSL-1, ligand for TLR2/6; 25 *μ*g/mL Poly I:C, ligand for TLR3; 100 ng/mL Flagellin, ligand for TLR5 (*Bacillus subtilis*); 5 *μ*g/mL Imiquimod, ligand for TLR7; 5 *μ*g/mL R848, ligand for TLR7 and TLR8; 6 *μ*g/mL CpG ODN 2216, ligand for TLR9 (all from Invivogen, San Diego, California, USA); 100 ng/mL LPS, ligand for TLR4 (*Escherichia coli* 055:B5; Sigma-Aldrich, St. Louis, MO); and heat-killed* Escherichia coli* (specific for TLR4; 1 : 10 mDC/bacteria ratio, Invivogen). TLR ligand concentrations used in the present paper were determined as optimal for DCs stimulation by preliminary experiments and previously published work by this group [13]. Where indicated, 10 *μ*M arachidonic acid was added. U0126 (a MEK1/2 inhibitor), PD98059 (an ERK1/2 inhibitor), SB203580 (a p38 MAPK inhibitor), JNK Inhibitor II (a JNK inhibitor), and BAY11-7082 (a NF-*κ*B inhibitor) were from Calbiochem (San Diego, CA).

### 2.3. PGE_2_ and CXCL8 Determination

DCs were incubated for 24 h with the indicated treatments. Cell-free supernatants were harvested and PGE_2_ production was measured by EIA (Cayman Chemical) kit. Secreted CXCL8 was measured by ELISA assay according to the manufacturer instructions (R&D Systems, Minneapolis, MN, USA).

### 2.4. Real-Time PCR

RNA was extracted in TRIzol, according to the manufacturer's instructions. After RNA purification, samples were treated with DNase to remove contaminating genomic DNA (DNaseI amplification grade). Reverse transcription was performed using random hexamers and Superscript II RT. All reagents were from Invitrogen. The iQ*™* SYBR Green Supermix (Bio-Rad Laboratories Inc., Hercules, CA, USA) for quantitative real-time PCR was used according to manufacturer's instructions. Reactions were run in triplicate on an iCycler*™* (Bio-Rad Laboratories Inc.) and the generated products analysed by the iCycler iQ Optical System Software (Version 3.0a, Bio-Rad Laboratories Inc.). Gene specific primers were as follows: hHPRT (forward: 5′-CCAGTAACAGGGGACATAAA-3′, reverse: 5′-CACAATCAAGACATTCTTTCCAGT-3′); hTLR1 (forward: 5′-CCTAGCAGTTATCACAAGCTCAAA-3′, reverse: 5′-TCTTTTCCTTGGGCCATTC-3′); hTLR2 (forward: 5′-CGTTCTCTCAGGTGACTGCTC-3′, reverse: 5′-CCTTTGGATCCTGCTTGC-3′); hTLR3 (forward: 5′-AGTTGTCATCGAATCAAATTAAAGAG-3′, reverse: 5′-AATCTTCCAATTGCGTGAAAA-3′); hTLR4 (forward: 5′-CTCCCCTGTACCCTTCTCACT-3′, reverse: 5′-CTCCCTGCCTTGAATACCTTC-3′); hTLR5 (forward: 5′-GACACAATCTCGGCTGACTG-3′, reverse: 5′-GCCAGGAACATGAACATCAA-3′); hTLR6 (forward: 5′-TGAAACAGTCTCTTTTGAGTAAATGC-3′, reverse: 5′-TCCATTTGGGAAAGCAGAGT-3′); hTLR7 (forward: 5′-TTAACCAATTGCTTCCGTGTC-3′, reverse: 5′-GGTGCCCACACTCAATCTG-3′); hTLR8 (forward: 5′-TGTGGTTGTTTTCTGGATTCAA-3′, reverse: 5′-GCTCGCATGGCTTACATGA-3′); hTLR9 (forward: 5′-TGTGAAGCATCCTTCCCTGT-3′, reverse: 5′-GAGAGACAGCGGGTGCAG-3′). Gene expression was normalized based on HPRT mRNA content.

### 2.5. SDS-PAGE and Western Blot

Following the designated treatments, DCs were washed twice with PBS and lysed in L1 buffer (50 mM Tris-HCl, pH 8.0; 2 mM EDTA; 0.1% NP-40 and 10% glycerol) with inhibitors to separate cytoplasmic proteins. Nuclear pellets were washed twice with L1 buffer with inhibitors and then lysed in NP-40 Lysis buffer (50 mM Tris-HCl, pH 8.0; 250 mM NaCl; 1 mM EDTA; 0.1% NP-40; and 10% glycerol) with inhibitors. Total cell extracts were obtained with NP-40 Lysis buffer. Equal amounts of cytoplasmic, nuclear, or total extracts were analysed through 8–12% SDS-PAGE followed by Western blotting with antibodies against COX2 (mouse monoclonal, Cat. 160112, Cayman Chemical), phospho-ERK1/2 (rabbit polyclonal, Cat. 9101, Cell Signalling Technologies, Massachusetts, USA), phospho-p38 (rabbit polyclonal, Cat. 9211, Cell Signalling), phospho-cPLA_2_ (rabbit polyclonal, Cat. 2831, Cell Signalling), phospho-MSK1 (rabbit polyclonal, Cat. 9595, Cell Signalling), NF-*κ*B p65 (rabbit polyclonal, C-20 Cat. sc-372, Santa Cruz Biotechnology), *β*-actin (mouse monoclonal, C-4 Cat. sc-44478, Santa Cruz Biotechnology), and Lamin B (goat polyclonal, C-20 Cat. sc-6216, Santa Cruz Biotechnology). Protein bands were detected with SuperSignal West Pico Chemiluminescent Substrate (Pierce, Rockford, USA). Densitometric analysis was performed using ImageJ (version 1.48) software package from National Institutes of Health. Immunoblots were scanned as JPEG images and the areas under the curves were measured for each band and quantified. Data were normalized based on *β*-actin or Lamin B content.

### 2.6. Release of [^14^C] AA

DCs (6 × 10^6^/mL, in RPMI 1640, 10% FCS) were labelled in Petriperm dishes with 0.125 *μ*Ci/mL [^14^C] AA (Amersham, Buckingham, UK) overnight. At the end of the incubation, cells were washed twice and resuspended in RPMI 1640 supplemented with 0.2% fatty acid free bovine serum albumin (Sigma). DCs were stimulated for 3 h and the reaction was terminated by the addition of 2 mL of chloroform/methanol/formic acid (1 : 2 : 0.2, v/v/v, all from Sigma-Aldrich) followed by agitation. Then, 1 mL of water and 2 mL chloroform were added. Chromatographic separation of lipids was performed by evaporating the organic phase under a stream of nitrogen, redissolving the residue in chloroform, and loading the extract on silica gel G plates (Merck, Darmstadt, Germany). Fatty acids were separated by thin layer chromatography using hexane/ethyl ether/formic acid (15 : 10 : 1, v/v/v, all from Sigma-Aldrich) as a solvent system for 30 min. AA position on TLC plates was determined as comigration with commercially available standard after exposure to iodine vapors. Autoradiography of TLC plates was performed using a phosphoimaging system (FLA 2000, Fuji). The results are expressed as the percentage of radioactivity in the arachidonic acid band on the total radioactivity recovered from each lane.

### 2.7. Immunohistochemistry

Formalin-fixed paraffin-embedded human tissues were retrieved from the archive of the Department of Pathology (Spedali Civili di Brescia, Brescia, Italy). Anti-PGE_2_ (rabbit polyclonal, 1 : 700 overnight, Biorbyt) was revealed using DakoEnvision + System-HRP Labelled Polymer Anti-Rabbit and DAB after antigen retrieval (thermostatic bath, TRIS-EDTA buffer, pH 9.0). Characterization of PGE_2_ positive cells was performed by double immunohistochemistry using CD11c (mouse, clone 5D11, 1 : 50, Leica Microsystems) and visualized using Mach 4 MR-AP (Biocare Medical, CA), followed by Ferangi Blue (Biocare Medical) as chromogen. Immunostained sections were photographed using the DP-70 Olympus digital camera mounted on the Olympus BX60 microscope.

### 2.8. Statistical Analysis

Statistical significance between the experimental groups was determined using one-way ANOVA with Dunnett's* post hoc* test (GraphPad Prism version 4.00 for Windows, GraphPad Software).

## 3. Results and Discussion 

### 3.1. The Stimulation of TLR4 and TLR7-8 Induces PGE_2_ in Human DCs

**Figure 1:**
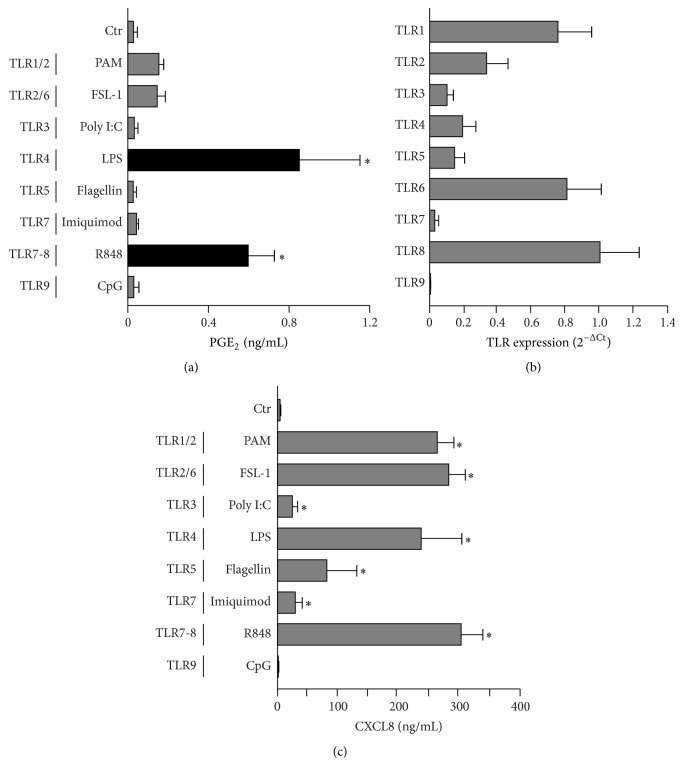
TLR4 and TLR7-8 stimulation induce the secretion of PGE_2_ by DCs. ((a) and (c)) DCs at day 6 of culture were stimulated with PAM_3_CSK_4_ (100 ng/mL), FSL-1 (100 ng/mL), Poly I:C (25 *μ*g/mL), LPS (100 ng/mL), Flagellin (100 ng/mL), Imiquimod (5 *μ*g/mL), R848 (5 *μ*g/mL), and CpG (6 *μ*g/mL). After 24 h, supernatants were collected and the production of PGE_2_ (a) and CXCL8 (c) was evaluated by EIA or ELISA, respectively. Results are expressed as mean ± SEM (*n* = 4); ^*∗*^ *P* < 0.05 compared with respective controls by one-way ANOVA with Dunnett's* post hoc* test. (b) mRNA from DCs at day 6 of culture was extracted to analyse the expression of TLRs. Data are expressed as mean ± SEM (*n* = 3).

Human DCs were stimulated with TLR-specific ligands and analysed for the release of PGE_2_.  Figure 1(a) shows that, in addition to LPS (TLR4 ligand), only R848 (TLR7 and TLR8 ligand, from now on TLR7-8) could stimulate the secretion of PGE_2_. The ligands for TLR1/2 (PAM_3_CSK_4_), TLR2/6 (FSL-1), TLR3 (Poly I:C), TLR5 (Flagellin), TLR7 (Imiquimod), and TLR9 (CpG) were by contrast ineffective. Thus, we asked whether these receptors were expressed and functional in DCs.  Figure 1(b) shows that DCs express all TLR mRNAs, exception made for TLR9. While the absence of TLR9 in myeloid DCs is generally recognized, the expression of TLR7 is controversial [14–16]. However, since TLR1–8 ligands activated DCs to produce CXCL8 (Figure 1(c)), we concluded that these receptors were indeed expressed and functional in our experimental setting. CpG was excluded from further analysis because of the lack of its cognate receptor.

Previous works have compared TLR ligands for their capability to induce differential cytokine production by human DCs [17–22]. To our knowledge, our study is the first to investigate eicosanoid production induced by different TLRs and to highlight so far oversight differential ability of TLR ligands to induce the release of PGE_2_ in human DCs.

### 3.2. MAPKs and NF-*κ*B Are Key Downstream Signalling Molecules for PGE_2_ Production

**Figure 2:**
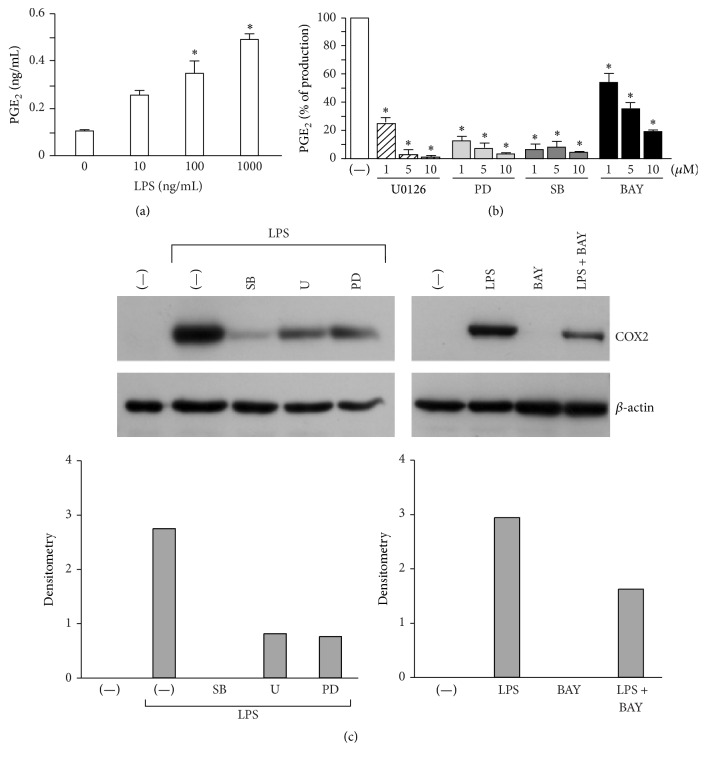
The release of PGE_2_ by DCs depends on the activation of the MAPK and NF-*κ*B pathways. (a) DCs were stimulated with increasing concentrations of LPS for 24 h and PGE_2_ production was quantified by EIA. Data are expressed as mean ± SEM (*n* = 3); ^*∗*^ *P* < 0.05 by one-way ANOVA with Dunnett's* post hoc* test. (b) DCs were pretreated for 1 h with the indicated doses of U0126, PD98059, SB203580, or BAY-11-7082 and then stimulated with LPS (100 ng/mL) for 24 h. The production of PGE_2_ was evaluated in cell-free supernatants by EIA. Results are expressed as mean ± SEM (*n* = 3); ^*∗*^ *P* < 0.05 by one-way ANOVA with Dunnett's* post hoc* test. (c) DCs were treated as in (b), using 1 *μ*M of each inhibitor. The expression of COX2 and *β*-actin was determined by Western blot. One representative fluorogram out of three and its densitometric analysis are shown.

LPS, a ligand inducing robust PGE_2_ secretion over a vast range of concentrations (Figure 2(a)), was used to further investigate the signalling pathways responsible for PGE_2_ production in DCs. Since in other experimental settings the regulation of PGE_2_ involves the activation of MAPKs and NF-*κ*B [3, 23, 24], DCs were stimulated in the presence of specific MAPK inhibitors such as U0126 (inhibitor of MAPK kinase), PD98059 (inhibitor of extracellular-signal-regulated kinase 1/2-ERK1/2), SB203580 (inhibitor of MAPK p38), and, of the NF-*κ*B inhibitor, BAY11-7082, which all significantly reduced the release of PGE_2_ (Figure 2(b)) when used at the lower concentration, without affecting cell viability (not shown). Of note, the same signalling pathways also regulated the induction of COX2, the rate-limiting enzyme for PGE_2_ synthesis (Figure 2(c)).

These results confirm that, in human DCs, the release of PGE_2_ depends on the activation of the MAPK and NF-*κ*B pathways.

### 3.3. TLR Ligands Differentially Activate MAPKs and NF-*κ*B in Human DCs

**Figure 3:**
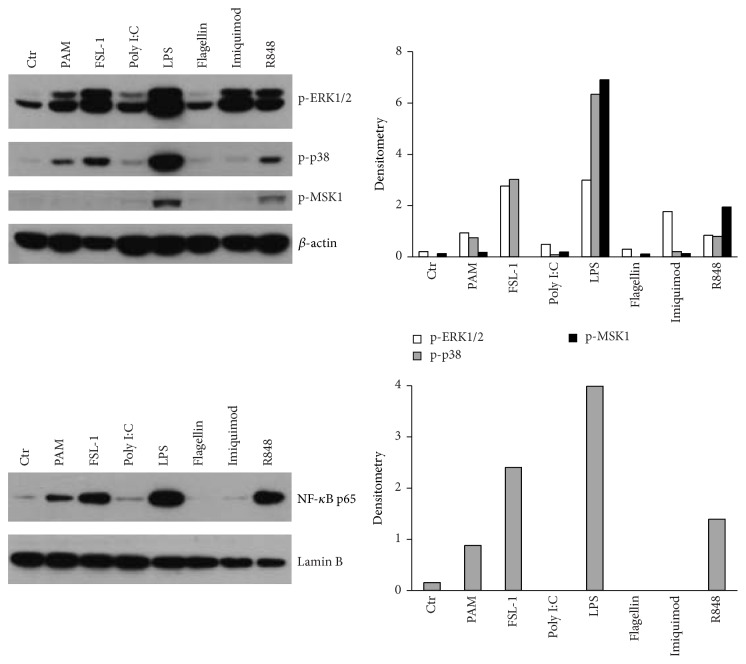
TLRs differentially activate the MAPK and NF-*κ*B pathways. DCs were stimulated with TLR agonists as indicated in Figure 1 for 30 min. After cell lysis, extracts were blotted against phospho-p38, phospho-ERK1/2, and phospho-MSK1. Nuclear extracts were blotted against NF-*κ*B p65. *β*-actin and Lamin B represent loading controls for total and nuclear proteins, respectively. The image depicts results obtained in one representative donor out of three.

In order to clarify the molecular mechanisms hindering the release of PGE_2_ by inactive TLR ligands, we analysed how different TLR stimulation impacted the activation of MAPKs and NF-*κ*B.  Figure 3 shows that all ligands induced ERK1/2 phosphorylation, although at different extent. However only LPS, R848, PAM_3_CSK_4_, and FSL-1 also induced p38 phosphorylation and NF-*κ*B p65 nuclear translocation, while Poly I:C, Flagellin, and Imiquimod did not. Finally, TLR2 ligands failed to phosphorylate MSK1, a kinase downstream ERK and p38 MAPK that was described to play a role in PGE_2_ production [25, 26]. Similar activation patterns were also detected at 15 and 60 minutes after stimulation (not shown).

Such striking differences in the activation of MAPKs and NF-*κ*B are interesting because, according to the literature, all TLR agonists are expected to converge on these pathways to exert their biological effects [27, 28]. Our results underline the importance to confirm and refine previous findings, obtained in model cell lines and often by transfection, in primary cells expressing TLRs at physiological levels.

Of particular interest was the difference in the activation induced by Imiquimod and R848, both in terms of intracellular signalling and in terms of PGE_2_ secretion. TLR7 and TLR8 both recognize ssRNA, are similar in sequence and localization, and, together with TLR9, form an evolutionary related TLR subfamily sharing common signalling pathways responsible for antiviral responses [28]. The differences we have observed may thus merely depend on the limited expression of TLR7, which would explain the lower PGE_2_ secretion induced by Imiquimod. However, Imiquimod phosphorylated ERK1/2 at levels that were comparable to, if not exceeding, those induced by R848, despite the fact that it failed to activate other signalling molecules. This may unveil a qualitative rather than a quantitative difference between the signalling pathways activated by TLR7 and TLR8, as suggested by other authors [20, 29]. In addition, we hypothesized that R848, by concomitantly triggering TLR7 and TLR8, may activate a synergy between the two signalling pathways. This issue represent an interesting area of investigation that will be intensively pursued.

### 3.4. TLR3, TLR5, and TLR7 Stimulation Fail to Induce COX2, While TLR1/2 and TLR2/6 Stimulation Fail to Mobilize AA

**Figure 4:**
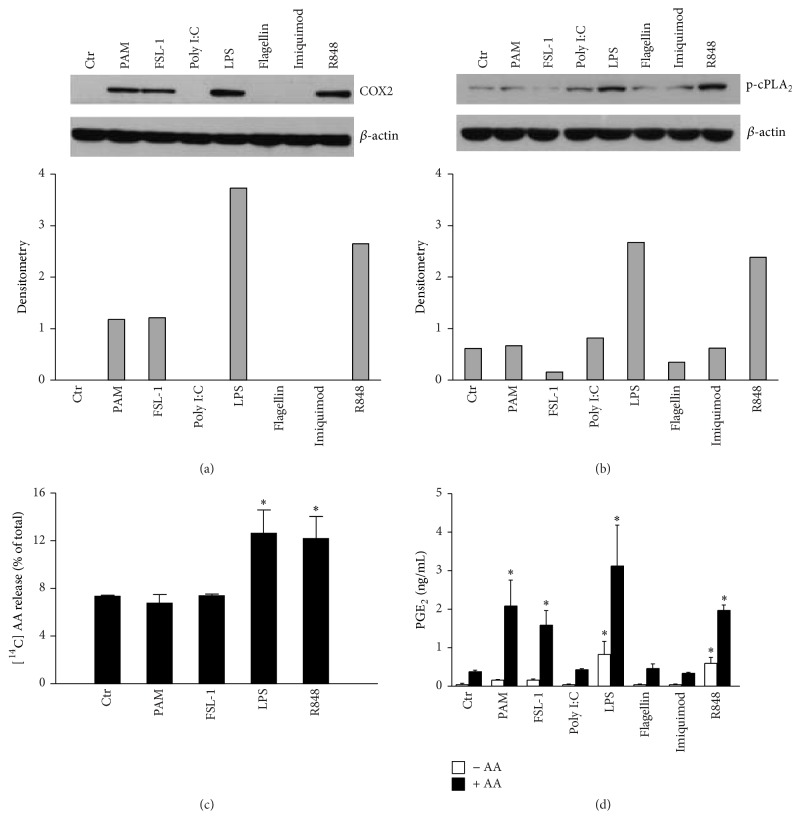
Lack of AA mobilization blocks the release of PGE_2_ upon TLR1/2 and TLR2/6 stimulation. (a) DCs were stimulated with TLR agonists for 24 h. The expression of COX2 and *β*-actin was determined by Western blot. Fluorogram from one out of 3 representative donors and its densitometric analysis are shown. (b) DCs were stimulated with TLR agonists for 30 min and the phosphorylation of cPLA_2_ was determined by immunoblot. One out of 3 representative donors and its densitometric analysis are shown. (c) DCs were labelled with 0.125 *μ*Ci/mL [^14^C] AA overnight and then stimulated with the indicated TLR ligands for 3 h. The results are expressed as the means ± SEM (*n* = 3) of the percentage of [^14^C] AA release on the total radioactivity recovered from each stimulation; ^*∗*^ *P* < 0.05 by one-way ANOVA with Dunnett's* post hoc* test. (d) DCs were incubated with TLR ligands in the presence (black bars) or absence (white bars) of 10 *μ*M AA. After 24 h, supernatants were collected and the production of PGE_2_ was evaluated by EIA. Results are expressed as mean ± SEM (*n* = 3); ^*∗*^ *P* < 0.05 by one-way ANOVA with Dunnett's* post hoc* test.

We next examined how the TLR-activated signalling pathways could differentially affect COX2 activation. Because COX2 activity directly correlates with protein levels [3], Western blot analysis was used to address this issue.  Figure 4(a) clearly shows that Poly I:C, Flagellin, and Imiquimod failed to induce COX2 accumulation, which fully explains the lack of PGE_2_ secretion and also confirms that NF-*κ*B activation is critical for COX2 expression [23, 24]. By contrast, PAM_3_CSK_4_ and FSL-1 were as effective as LPS or R848 in COX2 induction, suggesting that these ligands lack in downstream steps of PGE_2_ synthesis.

Thus, we analysed the activation of cPLA_2_, the other PGE_2_ key-producing enzyme. Within minutes, cPLA_2_ is regulated by phosphorylation [30]. We found that only LPS and R848 induced significant cPLA_2_ phosphorylation at 30 minutes after stimulation (Figure 4(b)). Based on the observations in Figure 3, it is tempting to speculate that, in our system, cPLA_2_ phosphorylation may depend on MSK1 activation, as previously demonstrated in human fibroblasts stimulated with IL-1*β* [25, 26].

Consistent with inefficient cPLA_2_ phosphorylation, PAM_3_CSK_4_ and FSL-1 did not induce AA release as compared to LPS and R848 (Figure 4(c)), suggesting that TLR1/2 and TLR2/6 stimulation may fail to induce PGE_2_ secretion because of the unavailability of AA, the substrate for COX2 enzyme. According to this hypothesis, the administration of exogenous AA (Figure 4(d), black bars) restored the production of PGE_2_ by PAM_3_CSK_4_ and FSL-1, but not by Poly I:C, Flagellin, and Imiquimod due to their inability to accumulate COX2.

Altogether, these results identify AA and COX2 accumulation as the PGE_2_-limiting checkpoints downstream TLR1/2-2/6 and TLR3-5-7 stimulation, respectively.

### 3.5. Human DCs Produce PGE_2_ *In Vivo*

Despite the fact that DCs are very well known PGE_2_ targets [2], their potential as prostaglandin sources in humans is less investigated and remains under debate. In fact, using* in vitro* differentiated DCs as a model, it was described that human DCs either could [31, 32] or could not produce PGE_2_ [33]. In addition, the strict ligand selectivity we have demonstrated so far raises the question of how primary DCs may respond to real pathogens in terms of PGE_2_ production.

**Figure 5:**
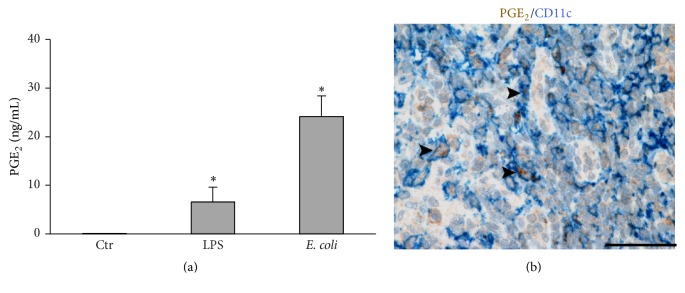
CD11c^+^ cells produce PGE_2_ in human inflamed lymph nodes. (a) Circulating mDCs were stimulated with LPS (100 ng/mL) or heat-killed* E. coli* (1 : 10 mDC/bacteria ratio). After 24 h, supernatants were collected and the production of PGE_2_ was evaluated by EIA. Data are expressed as mean ± SEM (*n* = 3); ^*∗*^ *P* < 0.05 by one-way ANOVA with Dunnett's* post hoc* test. (b) Sections from FFPE reactive lymph nodes were stained as indicated. Cytoplasmic PGE_2_ is observed in a fraction of CD11c^+^ cells. Representative double positive cells are indicated by arrow heads. Sections are counterstained with Meyer's haematoxylin. Original magnifications: 400x (scale bar 50 *μ*m).

To shed light on the possible role of DCs as PGE_2_-producing cells* in vivo*, we stimulated primary, circulating mDCs with TLR4 ligands.  Figure 5(a) shows that these cells respond to TLR4 stimulation releasing amounts of PGE_2_ that are much higher than those observed for their* in vitro*-derived counterparts. This is in line with previous observations that IL-4 used to generate DCs may hinder the activity of cPLA_2_ [33]. Finally, we performed double immunohistochemistry stainings on human inflamed lymph nodes, showing a fraction of CD11c^+^ cells expressing PGE_2_ in their cytoplasm (Figure 5(b)).

These results conform that primary DCs can actively secrete PGE_2_ in inflammatory conditions* in vivo*.

The net effect of the simultaneous stimulation of different TLRs and also of other classes of innate immune receptors by whole microorganisms remains to be elucidated. However, it is plausible that pathogens expressing molecular patterns activating TLR4 and TLR7-8 may be stronger PGE_2_ inducers. Thus, the pathogen-dictated modulation of the release of PGE_2_ may represent a novel mechanism through which DCs shape the immune and inflammatory responses.

## 4. Conclusion

**Figure 6:**
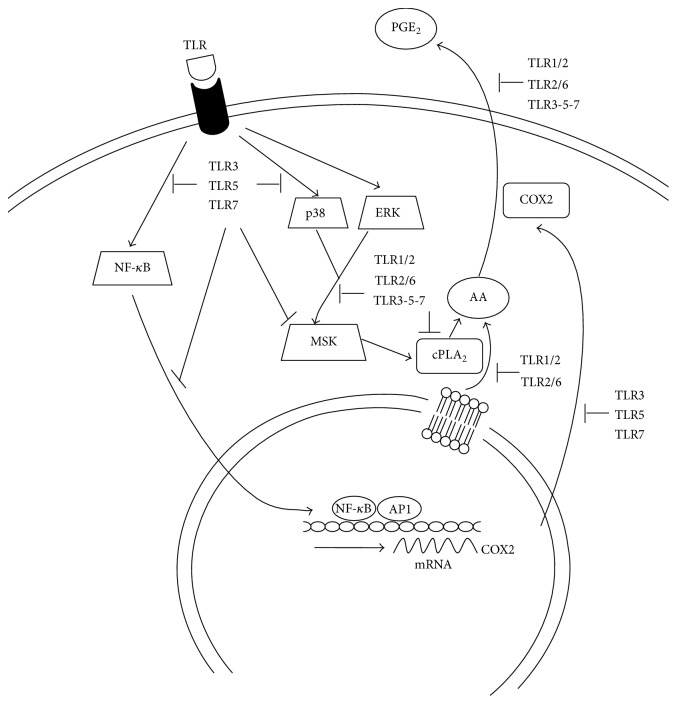
Mechanisms of PGE_2_ production by TLR family members. TLR4 triggering induces the transcription of COX2 via NF-*κ*B and MAPK p38 and ERK1/2 as well as cPLA_2_ phosphorylation and AA mobilization, presumably via MAPK and/or MSK1, which is in turn converted into PGE_2_ by COX2 and released into the medium. Similar mechanisms of action can be envisaged when TLR7 and 8 are concomitantly activated by R848. TLR3, TLR5, and TLR7 fail to activate NF-*κ*B and selected MAPKs, thus hindering the transcription of COX2. By contrast, TLR1/2 and TLR2/6 cause no PGE_2_ release because of inefficient cPLA_2_ phosphorylation and AA mobilization, which may correlate with their inability to phosphorylate MSK1.

In the present paper, we demonstrate a differential ability of TLR ligands to induce the release of PGE_2_ and provide a detailed description of the mechanisms governing TLR-mediated eicosanoid production in human DCs. A schematic representation of our findings is outlined in Figure 6. Briefly, only the stimulation of TLR4 and TLR7-8 could activate ERK1/2, p38, MSK1, and NF-*κ*B and induce PGE_2_. By contrast, the PGE_2_-limiting checkpoints downstream TLR1/2-2/6 and TLR3-5-7 stimulation were identified in AA and COX2 accumulation, respectively.

Our results also highlighted so far oversight differences in MAPK and NF-*κ*B activation by TLR ligands. These divergences may have come to light because, contrary to works performed in transfected cell lines, our system consisted of primary cells expressing a physiologic repertoire of receptors and intracellular adaptor molecules.

Finally, by demonstrating the expression of PGE_2_ by CD11c^+^ cells in human inflamed lymph nodes, this study further expands our knowledge on the complex role of DCs in the regulation of immune responses.
